# Intercellular Adhesion Molecular-5 as Marker in HIV Associated Neurocognitive Disorder

**DOI:** 10.14336/AD.2016.0918

**Published:** 2017-05-02

**Authors:** Lin Yuan, Feili Wei, Xin Zhang, Xianghua Guo, Xiaofan Lu, Bin Su, Tong Zhang, Hao Wu, Dexi Chen

**Affiliations:** ^1^Center for Infectious Disease, Beijing Youan Hospital, Capital Medical University, Beijing 100069, China; ^2^Beijing Institute of Hepatology, Beijing Youan Hospital, Capital Medical University, Beijing 100069, China; ^3^The Affiliated Hospital of Qingdao University, Organ Transplantation Center, Qingdao, Shandong 266003, China

**Keywords:** HIV, ICAM5, cognitive impairment, biomarker

## Abstract

Despite the use of antiretroviral drugs HIV associated neurocognitive disorders (HAND) are still common in HIV-seropositive patients. Identification of HIV patients with cognitive impairment in early-stage might benefit a great deal from disease progression monitoring and treatment adjustment. Intercellular adhesion molecule-5 (ICAM5), characteristically expressed on neuron, may suppress immune functions by inhibition of T cell activation in central nervous system. Previous studies have shown that ICAM5 could be detected in patients with brain injury. To investigate the relationship between cognitive impairment and ICAM5 in HIV patients, we compared soluble ICAM5 levels in paired CSF and plasma specimens from HIV-infected individuals with or without neurocognitive impairment. sICAM5 concentrations were measured by ICAM5 ELISA kit. A total of 41 Patients were classified into HIV infected with normal cognition (HIV-NC) and impaired cognition groups (HIV-CI) based on Memorial Sloan-Kettering Scale. CSF and plasma levels of sICAM5 in HIV-CI patients were significantly higher than HIV-NC group (*p*<0.0001, *p*=0.0054 respectively). sICAM5 concentrations in plasma strongly correlated with sICAM5 in CSF (r=0.7250, *p*<0.0001) and S100B in CSF (r=0.3812, *p*<0.0139). Among 6 follow-up patients we found that sICAM5 levels in CSF and plasma might change consistently with HAND progression. In summary, we have shown that the expressions of sICAM5 in CSF and plasma may correlate with neurocognitive impairment in HIV infected patients. The elevation of sICAM5 in plasma were correspond with that in CSF as a consequence of blood-brain barrier permeability changes. ICAM5 can serve as a potential and readily accessible biomarker to predict HIV associated neurocognitive disorder.

Human immunodeficiency virus (HIV) enters the central nervous system (CNS) early after infection and gives rise to HIV-associated neurocognitive disorder (HAND), which are characterized by a wild spectrum of behavioral, cognitive, and motor dysfunctions. The introduction of highly antiretroviral therapy (HAART) has dramatically declined the frequency of HIV-associated dementia (HAD), but the prevalence of milder form of HAND, asymptomatic neurocognitive impairment (ANI), might be 33% to 60% in HIV-seropositive people despite of successful antiretroviral therapy (ART). ANI is also associated with poor quality of life, unemployment, low adherence to drug regimens, and has higher risk of experience for symptomatic neurocognitive impairment or mild neurocognitive disorder (MND) [1-6]. Routine screening for cognitive difficulties in HIV-seropositive population might be benefit for discovering patients in early stage and performing subsequent management for HAND. Although comprehensive neuropsychological tests are appropriate for diagnosing HAND, they are unlikely to be adopted as a screening tool because of cost effective or time consuming [7, 8]. Many biomarkers in CSF or blood correlate with scores on neuropsychological tests but none has yet been developed for use in the clinics [9-16]. HAND-specific biomarker is still an urgent need to be investigated.

Persistent immune activation in CNS has been considered as one of the underlying pathogenic mechanisms of HAND, where neuron contact-dependent and contact-independent mechanisms involved in [17]. Neurons themselves may provide neuroprotective effects by regulation of cytokine synthesis in T cells through intercellular adhesion molecule-5 (ICAM5). ICAM-5 is characteristically expressed on the soma and dendrites of neuron. Through the ICAM5-CD11a/CD18 (LFA-1) interaction, Neurons diminish TCR dependent T-cell activation in CNS. Additionally, soluble ICAM-5 (sICAM5) derived from cleaved ICAM5 by activated T-cell or microglial-secreted matrix metalloproteinases-2 and-9 may compete with ICAM-1 and down-regulate T-cell activation through LFA-1 binding [18-24]. In our previous study, we verified that ICAM5 expressed on soma and neurite of neurons cultured in vitro, and neurons treated with matrix metalloproteinases (MMPs) became more vulnerable to HIV-1 gp120. These findings suggest that ICAM-5 may have the potential protective effect against HIV induced neuro-inflammation or neurodegeneration. sICAM5 might reflect the damage of vulnerable telencephalic neurons in HAND.

## MATERIALS AND METHODS

### Study subjects

Study subjects were enrolled from inpatients with HIV-1 infection in Henan and Yunnan provinces in China. None of the patients had symptoms or signs of active CNS disease or comorbidity. Cerebrospinal fluid (CSF) was acquired at the time of a clinically indicated lumbar puncture (LP) and plasma was taken simultaneously. LP and Intravenous blood sampling were performed referring to standard operating procedures. All the paired CSF and plasma specimens were centrifuged, and cell-free CSF and plasma were aliquot and stored at -80^°^C until the time of the assay. The Memorial Sloan-Kettering Scale (MSK) was used to categorize the neurocognitive impairment for each patient, which contains gradations that range from minor cognitive disturbance to profound and incapacitating disorders [25]. This study was approved by ethics committees at the Capital University of Medical Science in Beijing, and all participants provided informed consent.

**Table 1:** Demographic and disease characteristics of subjects

Characteristics	HIV-CI	HIV-NC	*p*-value
**No. of Subjects**	19	22	
**Age in years [median (IQR)]**	44 (40-47)	40(24-45)	
**Gender [n (%)]**			
**Male**	12 (63%)	14 (64%)	
**Female**	7 (37%)	8 (36%)	
**HIV RNA (log10 copies/ml) [median (IQR)]**			
**CSF**	3.50 (2.05-5.50)^a^	2.56 (1.08-3.60)^b^	*p*=0.4460
**Plasma**	5.24 (2.74-20.50)^c^	4.10 (2.11-5.61)^d^	*p*=0.7187
**CD4+ cell count (cell/μl) [median (IQR)]**	80(32-184)	90 (27-234)	*p*=0.9646
**CSF**			
**cell count (cell/μl) [median (IQR)]**	36 (10-120)	20 (10-25)	*p*=0.0355
**protein (mg/L) [median (IQR)]**	1300 (700-2360)	400 (275-700)	*p*=0.0005
**No. of threated by HAART [n (%)]**	12 (63%)	15 (68%)	
**Treatment duration (months) [median (range)]**	4 (2-6)	6 (2-24)	

a *n* =11 samples.

b *n* =8 samples.

c *n* =15 samples

d *n* =18 samples

### ICAM5 and S100B measurement

The concentrations of ICAM5 and S100B in CSF or plasma were determined by ICAM5 ELISA kit (Cloud-Clone Corp. Houston, TX, USA; catalog #SEA142Hu.) and S100B ELISA kit (BioVendor-Laboratorní medicína a.s., Karasek, Brno, Czech Republic, Catalog #RD192090100R.) respectively. Specimen preparation and assay procedure were performed strictly according to the manufacturer’s instructions. The lower limit of detection (LLD) of ICAM5 was 1.56 ng/mL, and that of S100B was 10 pg/mL. All specimens were assayed concurrently in duplicate. Data were collected using xMark™ microplate spectrophotometer (Bio-Rad Laboratories Inc., Hercules, California, USA). Concentrations lower than LLDs were reported as undetectable (0)

### Statistical Analyses

Statistical analyses were performed and graphs constructed using GraphPad Prism 5 (GraphPad Software, San Diego, California, USA). The variables were compared independently by Mann-Whitney’s test. Spearman rank tests were used to test for correlations. *P* values less than 0.05 were considered significant.

**Figure 1.**
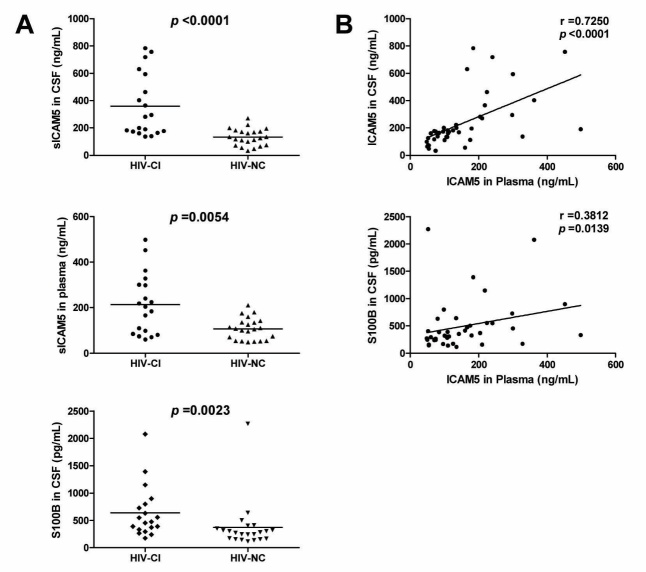
Comparison of sICAM5 and S100B concentrations in paired CSF and plasma samples from 19 HIV-CI and 22 HIV-NC patients The concentrations of sICAM5 whether in CSF or plasma samples from HIV-CI group were significantly higher than HIV-NC group. Additionally, the CSF levels of S100B were higher in HIV-CI patients **(A)**. Plasma levels of sICAM5 were correlated with CSF levels of sICAM5 or S100B **(B)**. Differences were analyzed by the Mann-Whitney U test. Spearman rank test was used for correlation analysis. *p* values of <0.05 were considered significant. Dots, sICAM5 or S100B concentrations in CSF or plasma for each study subject; horizontal lines, median values for each group.

## RESULTS

A total of 47 paired CSF and plasma specimens from 41 patients infected by HIV-1 clade B or B’ were analyzed. This included 6 patients with multiple samplings: four sampled twice and two sampled three times. Patients were predominantly men and middle-aged, had been diagnosed with HIV-1 for nearly a decade. The risk factors for HIV infection were blood transfusion (n=15), paid blood or plasma donation (n=12), sexual transmission (n=6), intravenous drug use (n=1), mother-to-child transmission (n=1) and unknown (n=6). Over 60 percent of the patients were on HAART, which consisted of at least two NRTIs (e.g., AZT, D4T, 3TC, DDI, and TDF) plus a NNRTI (NVP or EFV) or a PI (LPV). None were on mono or dual therapy. Based on MSK classification, patients were classified into HIV infected with normal cognition (HIV-NC; n=22, MSK=0) and impaired cognition groups (HIV-CI; n=19). HIV-CI group consisted of MSK=0.5 (n=10), MSK=1 (n=2), MSK=2 (n=3), and MSK=3 (n=4). Their blood CD4+ T cells were relatively low. The HIV-1 RNA concentrations in plasma were available for 80%, whereas that in CSF were only available for 46%. There was no significant difference in CSF or plasma HIV-1 RNA concentration between these groups, as well as in blood CD4+ T cell counts. CSF cell count and protein concentration in HIV-CI group were significantly higher than those in HIV-NC group (*p*=0.0355, *p*=0.0005 respectively) (Table 1).

**Figure 2.**
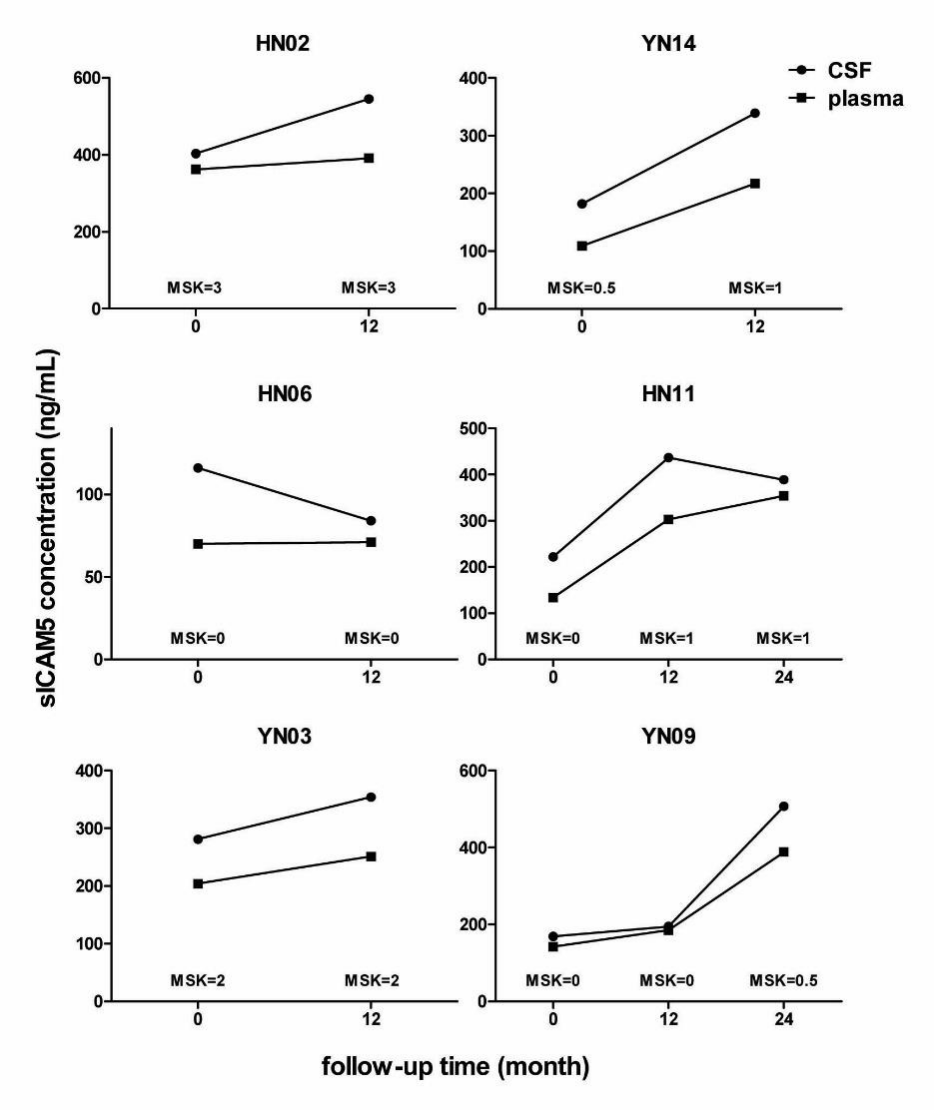
sICAM5 concentrations in CSF and plasma The changes of sICAM5 in CSF and plasma samples from 6 follow-up HIV infected individuals are shown. In patients YN14, HN11 and YN09, the concentrations of sICAM5 in CSF and plasma elevated significantly along with the increase of MSK score, while in patients HN02, HN06 and YN03, whose MSK scores were relatively stable, the changes of sICAM5 concentration in CSF or plasma were not significant. CSF values are shown in circles and plasma values are shown in squares.

There were significant differences in CSF and plasma levels of ICAM5 between HIV-CI and HIV-NC group (*p* < 0.0001, *p* = 0.0054 respectively). S100B levels in CSF were significantly higher in HIV-CI patients (*p* = 0.0023) (Fig. 1A). Plasma ICAM5 concentration correlated with CSF ICAM5 (r=0.7250, p<0.0001) and CSF S100B (r=0.3812, *p*<0.0139) (Fig. 1B).

We analyzed specimens from multiple time points from 6 patients. Significantly elevated levels of ICAM5 in CSF and plasma were shown along with disease progressing from HIV-NC to HIV-CI in three of these 6 patients, while in rest of three patients who stood still in relative normal cognitive conditions, the changes in CSF or plasma ICAM5 were not significant. Additionally, the change of ICAM5 in plasma was always correspond with that in CSF in any case (Fig. 2).

## DISCUSSION

The current approaches to identify or classify cognitive impairment in HIV-infected patients are mainly based on comprehensive neuropsychological testing. Many biomarkers correlate with scores on cognitive testing have been evaluated as screening tools for diagnosis and prognostication of HAND, but none have yet been developed for clinical use. ICAM5 is a specific cell adhesion molecule which is characterized expressed in the soma-dendritic membrane of neurons in the mammalian telencephalon. ICAM5 participates in formation of neuronal synapse and immunological synapse. It may compete with ICAM-1 to bind to an inhibitory site on LFA-1 and downregulate the TCR mediated T-cell activation. ICAM5 can be cleaved under several pathological conditions in the brain and its soluble form sICAM5, known as telencephalin, is strictly CNS tissue origin. sICAM5 has been detected in experimental hypoxic-ischemic brain injury, traumatic brain injury, temporal lobe epilepsy, Alzheimer’s disease and herpes simplex encephalitis [26-33]. Immunoassay on sICAM5 may turn out to be clinically useful in the diagnosis of brain diseases. To our knowledge, there is no studies on association between CSF or plasma ICAM-5 levels and neuro-cognition in HAND. Chronic immune activation in the CNS during HIV infection may give rise to neuronal ICAM5 cleavage by T cell induced MMPs and release of sICAM5 into CSF and blood. To distinguish HAND from other causes of neuro-inflammation, patients enrolled in our present study were excluded by clinical reports from other common CNS comorbidities, such as cryptococcal meningitis, tuberculous meningitis, encephalitis, toxoplasma encephalitis, progressive multifocal leukoencephalopathy, cytomegalovirus radiculitis or HBV/HIV coinfection. It was shown that ICAM5 levels in CSF and plasma specimens from HIV infected patients with neurocognitive impairment were significantly higher than those with normal neurocognitive. In several cases, development of cognitive disorder was accompanied by elevated levels of ICAM5 in CSF and plasma. These findings suggest that ICAM5 might be a useful biomarker for predicting HAND.

S100B is secreted by astrocytes or can spill from injured cells and enter the extracellular space or bloodstream. S100B has emerged as a candidate peripheral biomarker of blood-brain barrier (BBB) permeability and CNS injury [34-36]. In present study, the concentrations of sICAM5 in plasma were correlated with S100B levels in CSF. S100B levels were significantly elevated in CSF specimens of HIV-CI patients, but not in plasma (data not shown). However, more importantly, the changes of sICAM5 in plasma were strongly correlated with that in CSF, which might be the consequence of BBB permeability changes indicated by S100B. Readily available in blood specimens make sICAM5 better adopted for clinical screening of HAND.

The mechanism for development of cognitive impairment in HAND patients is not well understood. Although sICAM5 correlated with MSK scores on cognitive, its association with neuronal impairment of brain parenchyma has not yet determined. In this study, 46% of the patients had detectable HIV-1 RNA in the CSF at screening by standard clinical assays, with 11 of 19 in HIV-CI group and 8 of 22 in HIV-NC group. Further work is needed to identify the impact of HIV RNA in CSF on neurocognitive impairment and the expression of sICAM5 by neuron. Likewise, over 60% of enrolled patients were treated with cART containing at least three antiretroviral drugs. Whether antiretroviral drugs cause persistent cognitive impairment has not been well studied. The association between concentrations of antiretroviral drug and ICAM5 in CSF need further study. In addition, larger scales of cross-sectional and longitudinal studies are needed to examine the potential of ICAM5 in predicting HAND.

In conclusion, in this study, we showed that sICAM5 in CSF and plasma specimens from HIV-CI patients were significantly higher than HIV-NC patients. Levels sICAM5 in plasma were strongly correlated with that in CSF. sICAM5 can serve as a potential and readily accessible biomarker to predict HIV associated neurocognitive disorder.
